# Rethinking the evolution of eukaryotic metabolism: novel cellular partitioning of enzymes in stramenopiles links serine biosynthesis to glycolysis in mitochondria

**DOI:** 10.1186/s12862-017-1087-8

**Published:** 2017-12-04

**Authors:** Melania Abrahamian, Meenakshi Kagda, Audrey M. V. Ah-Fong, Howard S. Judelson

**Affiliations:** Department of Microbiology and Plant Pathology, University of California, Riverside, CA 92521 USA

**Keywords:** Compartmentalization of metabolism, Mitochondria, Glycolysis, Serine metabolism, Oomycete, Horizontal gene transfer

## Abstract

**Background:**

An important feature of eukaryotic evolution is metabolic compartmentalization, in which certain pathways are restricted to the cytosol or specific organelles. Glycolysis in eukaryotes is described as a cytosolic process. The universality of this canon has been challenged by recent genome data that suggest that some glycolytic enzymes made by stramenopiles bear mitochondrial targeting peptides.

**Results:**

Mining of oomycete, diatom, and brown algal genomes indicates that stramenopiles encode two forms of enzymes for the second half of glycolysis, one with and the other without mitochondrial targeting peptides. The predicted mitochondrial targeting was confirmed by using fluorescent tags to localize phosphoglycerate kinase, phosphoglycerate mutase, and pyruvate kinase in *Phytophthora infestans*, the oomycete that causes potato blight. A genome-wide search for other enzymes with atypical mitochondrial locations identified phosphoglycerate dehydrogenase, phosphoserine aminotransferase, and phosphoserine phosphatase, which form a pathway for generating serine from the glycolytic intermediate 3-phosphoglycerate. Fluorescent tags confirmed the delivery of these serine biosynthetic enzymes to *P. infestans* mitochondria*.* A cytosolic form of this serine biosynthetic pathway, which occurs in most eukaryotes, is missing from oomycetes and most other stramenopiles. The glycolysis and serine metabolism pathways of oomycetes appear to be mosaics of enzymes with different ancestries. While some of the noncanonical oomycete mitochondrial enzymes have the closest affinity in phylogenetic analyses with proteins from other stramenopiles, others cluster with bacterial, plant, or animal proteins. The genes encoding the mitochondrial phosphoglycerate kinase and serine-forming enzymes are physically linked on oomycete chromosomes, which suggests a shared origin.

**Conclusions:**

Stramenopile metabolism appears to have been shaped through the acquisition of genes by descent and lateral or endosymbiotic gene transfer, along with the targeting of the proteins to locations that are novel compared to other eukaryotes. Colocalization of the glycolytic and serine biosynthesis enzymes in mitochondria is apparently necessary since they share a common intermediate. The results indicate that descriptions of metabolism in textbooks do not cover the full diversity of eukaryotic biology.

**Electronic supplementary material:**

The online version of this article (10.1186/s12862-017-1087-8) contains supplementary material, which is available to authorized users.

## Background

An important feature of the eukaryotic cell that arose during evolution is metabolic compartmentalization [1]. The partitioning of reactions between the cytosol and mitochondria became possible after the latter organelle evolved through endosymbiosis [2]. Similar possibilities arose after other organelles such as peroxisomes evolved [3]. A textbook example of metabolic partitioning is the division of glycolysis and the Krebs (tricarboxylic acid) cycle between the cytosol and mitochondria, respectively. Partitioning poses several potential advantages including reducing futile cycling, separating reactions that occur optimally at different pH, and increasing reaction velocities by raising the local concentration of substrates. Such benefits are balanced by the need to transport metabolites between compartments.

While glycolysis is normally defined as a cytosolic process, there is some diversity within eukaryotes. For example, some kinetoplastid protozoans encase glycolytic enzymes in a peroxisome-like organelle called the glycosome [4]. In plants, certain glycolytic enzymes reside both in the cytosol and the plastid, where some reactions are shared with photosynthesis [5]. Another example of diversity is the use by some bacteria, protists, and plants of pyrophosphate (PPi) instead of ATP as the phosphate donor in the initial “preparatory stage” of glycolysis, where six-carbon sugars are converted to two triose phosphates [6]. Such interspecific differences reflect both lineage-specific innovations and acquisitions through events such as horizontal gene transfer.

Exceptions to the paradigm of cytosolic glycolysis have been proposed in the stramenopiles [7, 8]. This eukaryotic lineage, also known as heterokonts due to their two distinct flagella, includes “colored” or photosynthetic groups such as diatoms and brown algae, and non-photosynthetic taxa such as oomycetes and the animal parasite *Blastocystis* [9]. Whether oomycetes and *Blastocystis* diverged prior to the acquisition of plastids by the photosynthetic lineages, or if oomycetes lost their plastids later during evolution, has been debated [10, 11]. Remarkably, bioinformatic studies of oomycete and diatom genomes predicted that enzymes from the last half of glycolysis, from triose phosphate isomerase to pyruvate kinase, occur as both canonical cytosolic forms and those that contain mitochondrial targeting peptides [8, 12–14]. The targeting peptides are N-terminal amphipathic helices that are recognized by the mitochondrial protein import pathway [15]. The unusual enzymes constitute the “payoff-phase” of glycolysis, which generates ATP and intermediates for other pathways including lipid and amino acid biosynthesis [16].

The goals of this study were to validate the predicted mitochondrial locations of the enzymes and reveal physiological or evolutionary explanations for their unusual location. This was achieved using *Phytophthora infestans,* a member of the oomycete group of the stramenopiles. *P. infestans* causes the late blight disease of potato, which triggered the Irish Famine in the mid-1800’s and still limits crop production [17]. Using fluorescently tagged proteins, we were able to show that the payoff-phase enzymes truly reside in mitochondria. A search for other atypically mitochondrial enzymes identified phosphoglycerate dehydrogenase (PGDH), phosphoserine aminotransferase (PSAT), and phosphoserine phosphatase (PSP), which comprise a pathway that converts the glycolytic intermediate 3-phosphoglycerate to serine. In non-stramenopiles, these are cytosolic [18, 19]. Phylogenetic analyses suggested that the unusual enzymes might have originated through modifications of previously cytoplasmic proteins acquired by descent and by ancient horizontal or endosymbiotic gene transfer events. Lateral transfer was also suggested by observations that genes encoding some of the unusual glycolytic enzymes and the serine metabolism pathway were adjacent to each other on oomycete chromosomes.

## Results

### Overview of glycolysis

**Fig. 1:**
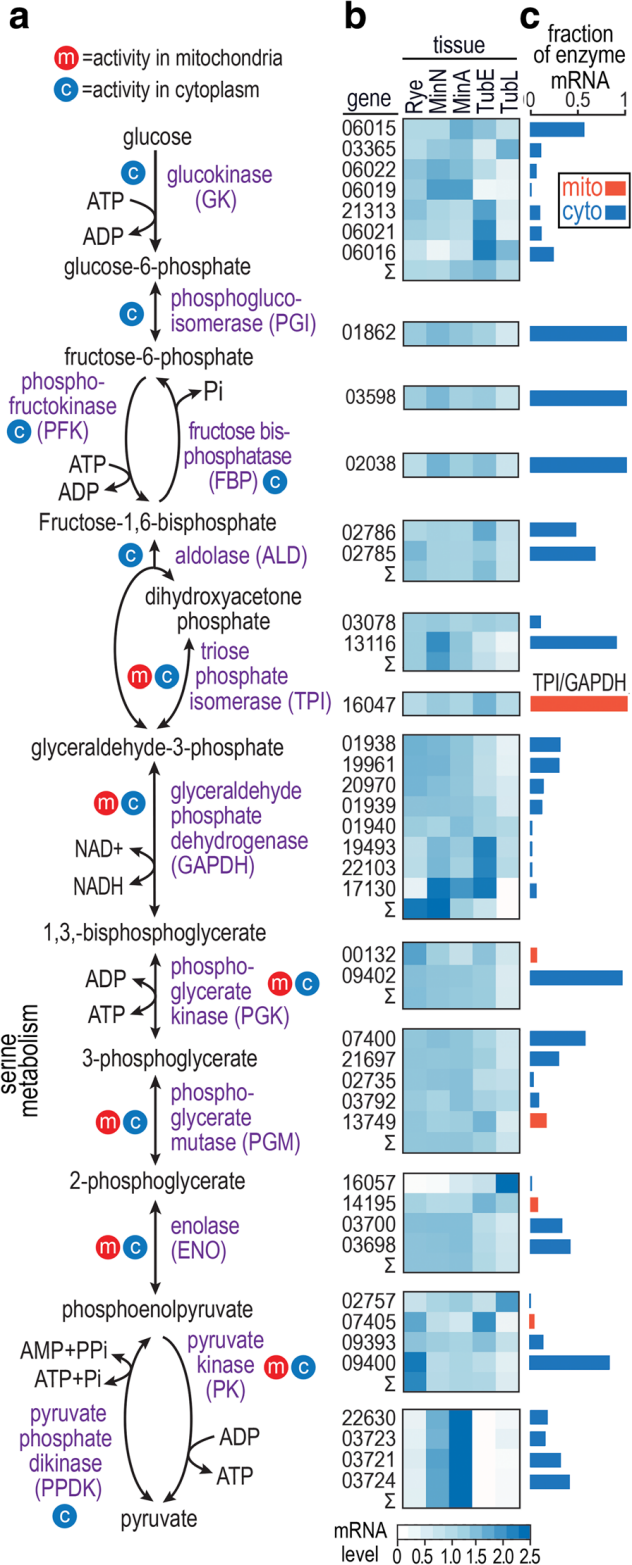
Glycolysis in *Phytophthora infestans.* **a,** Overview. Metabolites are indicated by black text and enzymes are indicated by purple text. Enzymes having mitochondrial or cytoplasmic forms are marked by red and blue circles, respectively. **b,** mRNA levels during growth on complex media (rye grain media), minimal media with glucose or amino acids as the main carbon source (MinA, MinN), and potato tubers at 1.5 and 4 days post-infection (TubE, TubL). Data are from RNA-seq analysis and are shown as per gene-normalized CPM values after TMM normalization by edgeR. Heatmaps are to the right of the corresponding enzymes in panel **a**. The five-digit numbers on the left side of the heatmap are the gene names, trimmed of the “PITG_” prefix. For enzyme activities produced by more than one gene, the sum of CPM values in each tissue type is represented by the row labeled Σ. **c,** Contribution of each gene to the total transcript pool for each enzyme, based on RPKM values. For example, the bar for glucokinase gene 06015 equals 0.54, which means that its transcripts represent 54% of all mRNAs encoding glucokinase. Mitochondrial and cytoplasmic forms of each enzyme are represented by red and blue bars, respectively

**Fig. 2:**
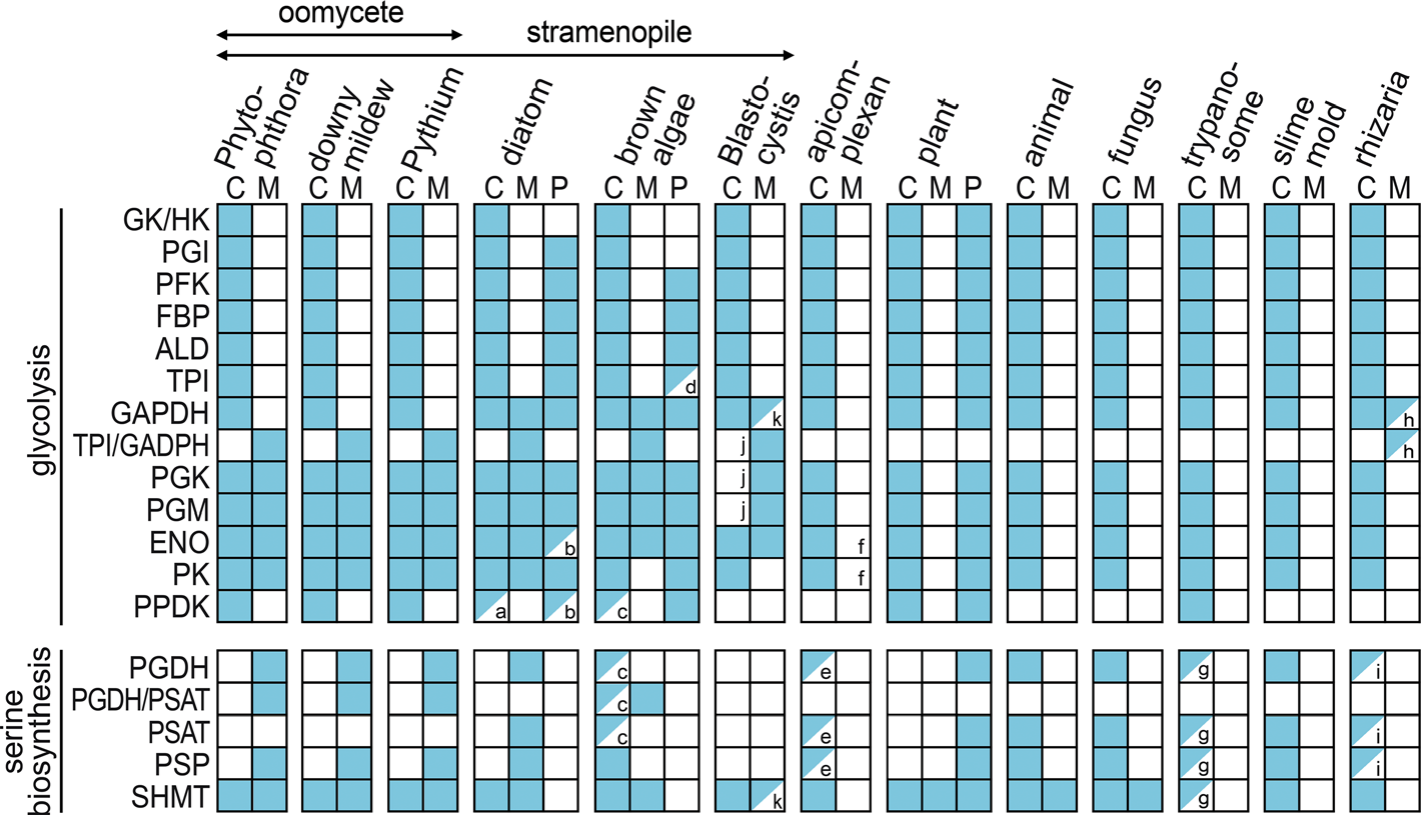
Summary of locations predicted for glycolytic and serine metabolism enzymes. Activities are marked as cytosolic (C), mitochondrial (M), or plastidic (P). Filled squares indicate that >75% of species within the group contain an enzyme with the indicated location, while half-filled squares denote between 25% and 75% of species. The 72 species represented by the table are listed in Additional file 2, and the accession numbers of the sequences are in Additional file 3. Notes within boxes are a, in *T. pseudonana* and *F. cylindrus* but not *P. tricornutum*; b, in *F. cylindrus* and *P. tricornutum* but not *T. pseudonana*; c, in *Cladosiphon okamuranus* but not *E. siliculosus*; d, in *E. siliculosus*, not *C. okamuranus;* e, missing in *Plasmodium* spp.; f, possible mitochondrial protein in *Neospora caninum;* g, in *Leishmania* and *T. cruzi* but not *T. brucei* or *T. vivax;* h, found in *Paulinella chromatophora* but not *Bigelowiella natans, Plasmodiophora brassicae,* or *Reticulomyxa filosa;* i, present in *P. brassicae,* and *R. filosa* but absent from *P. chromatophora* and *B. natans;* j, based on revised gene models, although note that the targeting results are ambiguous for the TPI-GAPDH fusion in subtype 4 since the scaffold terminates near the 5′ end of the gene; and k, present only in *B. hominis* and *Blastocystis* subtype 1

Fig. 1a outlines the steps of glycolysis in *P. infestans*. Nine of the 13 enzyme activities are encoded by multigene families, as shown in Fig. 1b where the five digit numbers represent gene identifiers, trimmed of their PITG prefixes. Figure 1b and c also display the patterns and levels of expression of each gene based on RNA-seq analysis of growth in rye media, minimal media, and potato tubers. A summary of the enzymes present in oomycetes *(Phytophthora, Pythium,* downy mildews), other stramenopiles (diatoms, brown algae, *Blastocystis*), and other eukaryotes is shown in Fig. 2.

**Fig. 3:**
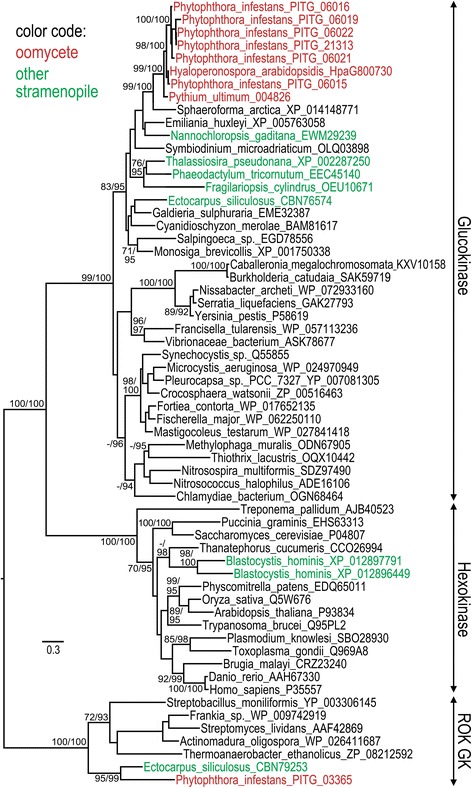
Phylogenetic analysis of glucokinases and hexokinases. Trees were generated using PhyML as described in Methods. Numbers at nodes represent bootstrap values above 70% from PhyML, and posterior probability (PP) values above 90 from mrBayes. Oomycetes are shown in red and other stramenopiles in green. Each sequence is marked with its GenBank accession number, except for oomycete proteins which use gene numbers assigned by their respective genome projects. Whether the proteins match pfam domains for glucokinase, hexokinase, or ROK glucokinases is indicated in the right margin

While the first step in glycolysis in most eukaryotes involves hexokinase (EC 2.7.1.1; KEGG orthology number K00844), this has been replaced by glucokinase (EC 2.7.1.2; K00845) in *P. infestans* and other stramenopiles with the exception of *Blastocystis.* As shown in Fig. 3, six of the enzymes from *P. infestans* and those from the downy mildew *Hyaloperonospora arabidopsidis* and *Pythium ultimum* form a well-supported clade with bacterial glucokinases. Also in the glucokinase clade are enzymes from the diatoms *Fragilariopsis cylindrus, Phaeodactylum tricornutum,* and *Thalassiosira pseudonana* and the brown alga *Ectocarpus siliculosus.* In contrast, the stramenopilian animal parasite *Blastocystis hominis* only encodes hexokinases. The oomycete and diatom enzymes show greater sequence similarity to glucokinases from cyanobacteria compared to other bacteria. For example, *P. infestans* enzyme PITG_06022 has up to 60% amino acid similarity to many cyanobacterial enzymes (e.g. *Cyanothece* spp. PC8801, Genbank accession ACK66739.1) compared to 51% against proteins from other bacterial groups (e.g. *Nannocystis exedens* SFD47329.1). In the tree shown in Fig. 3, however, the clade bearing cyanobacterial enzymes is not obviously closer to stramenopiles than other bacteria. *P. infestans* and *Ectocarpus* also encode ROK glucokinases (pfam00480; no corresponding Kegg orthology number), which were identified originally in bacteria as a family of carbohydrate-responsive transcriptional repressors and sugar kinases [20].

**Fig. 4:**
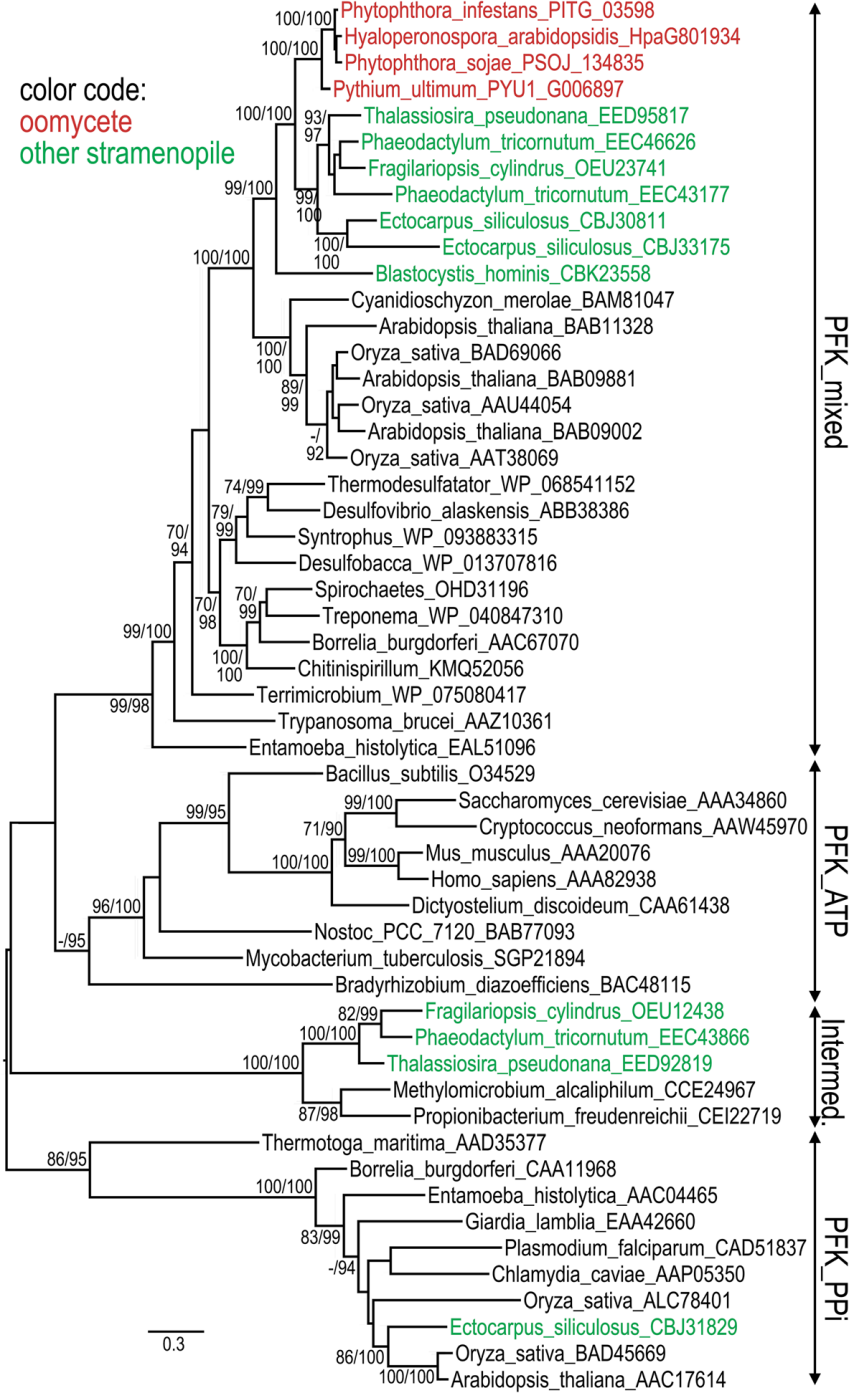
Phylogenetic analysis of phosphofructokinases. Trees were constructed as described in Fig. 3. Whether the sequences match protein domains for ATP, PPi (PPi), or mixed PFK is indicated in the right margin. The group labeled “Intermediate” includes enzymes that have only marginally better matches against the PFK_mixed than PFK_ATP domains

Oomycetes have also been reported to diverge from the classic form of eukaryotic glycolysis by expressing phosphofructokinases, PFKs, that use PPi (EC 2.7.1.90, K00895) instead of ATP (EC 2.7.1.11, K00850) as the phosphoryl donor [21]. However, this assignment of substrate specificity may be premature. PFKs can be placed into three categories as illustrated by the phylogenetic tree in Fig. 4. The first, defined by the PFKA_ATP (TIGR02482) domain, are ATP-utilizing and typical of most non-plant eukaryotic PFKs. The second, defined by the PFKA_PPi (TIGR02477) domain, is PPi-utilizing and found commonly in plants, anaerobic protists, and anaerobic bacteria. All oomycete, diatom, and *Blastocystis* PFKs cluster in the third group, defined by PFKA_mixed (TIGR02483), which also includes enzymes from bacteria, plants, and some protists. Few PFKA_mixed enzymes have been studied biochemically, but some have been shown to use ATP [22, 23] and others PPi [24]. Most stramenopilian PFKs are clearly diverged from the canonical ATP-utilizing enzymes of animals and fungi, but whether the *P. infestans* enzymes are truly PPi-utilizing remains to be demonstrated. In plants, biochemical studies have suggested that many PPi-dependent PFKs have been misannotated in terms of their substrate [23].

Oomycetes do encode a second PPi-utilizing enzyme: pyruvate, phosphate dikinase, PPDK (EC 2.7.9.1; K20115). This enzyme interconverts phosphoenol pyruvate and pyruvate at the last step of glycolysis, and is common in plants and bacteria [21]. Oomycetes also encode pyruvate kinase (PK), which catalyzes the same reaction. Since the reaction catalyzed by PPDK is more reversible than the PK reaction, the presence of PPDK may facilitate gluconeogenesis [25].

The most striking divergence from classical glycolysis becomes evident when the subcellular localization of the enzymes is considered. In *P. infestans* and other oomycetes, phosphoglycerate kinase (PGK), phosphoglycerate mutase (PGM), enolase (also known as phosphopyruvate hydratase; ENO), and pyruvate kinase (PK) are predicted to be expressed in both cytoplasmic and mitochondrial forms from separate genes, based on analyses using the TargetP and Mitofates programs [26, 27]. The enzymes with mitochondrial and/or cytoplasmic forms are labeled in Fig. 1a with red and blue icons, respectively, and the targeting prediction scores are shown in Additional file 1: Table S1. For example, genes PITG_09402 and PITG_00132 are predicted to encode cytoplasmic and mitochondrial PGK, respectively. This is consistent with prior studies focused on diatoms [8, 11, 13, 14]. Also predicted to be mitochondrial is a gene fusion encoding triose phosphate isomerase and glyceraldehyde phosphate dehydrogenase, TPI-GAPDH. Therefore, enzyme activities representing the entire second half of glycolysis may occur in two locations.

### Predicted mitochondrial payoff-phase enzymes are expressed

Since the predicted mitochondria-targeted glycolytic enzymes might be evolutionary relics such as pseudogenes, we obtained RNA-seq data to check for evidence of transcription*.* Transcripts were detected (CPM > 2) for all cytosolic and mitochondrial forms of the glycolytic enzymes during growth on rich rye media, minimal media, or tubers at early and late stages of infection. As shown in the heatmaps in Fig. 1b, expression patterns varied within most gene families. For example, mitochondrial PGM and ENO had higher mRNA during early tuber infection, while their cytoplasmic forms had low mRNA levels in that stage.

Within gene families, there was major variation in the level of transcription of each gene. This is illustrated by bar graphs in Fig. 1c. The mRNA levels of the genes encoding mitochondrial proteins (red bars) averaged about 5% of their cytosolic counterparts (blue bars). In most cases, the expression of the genes encoding mitochondrial proteins did not influence strongly the overall pattern of expression of each gene family, which is represented by the Σ rows in Fig. 1b. For example, even though the mitochondrial PGM peaked in early tuber infection, the aggregate expression (Σ) of all PGM genes was stronger in the three artificial media.

**Fig. 5:**
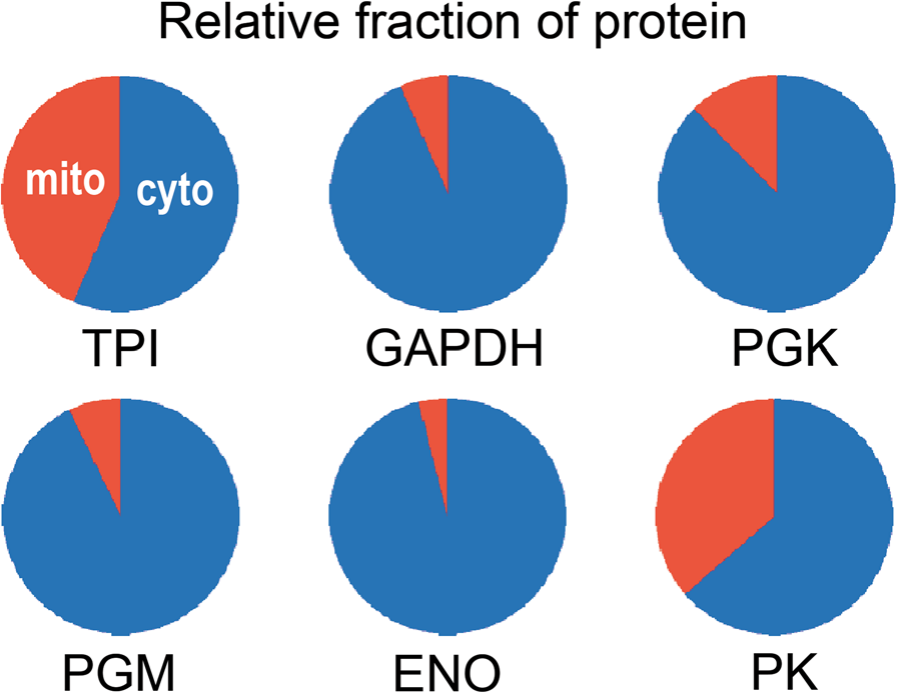
Fraction of protein with cytosolic versus mitochondrial localization. Values are based on LC-MS/MS and approximated using the emPAI method as described in Methods. TPI and GAPDH values include the TPI-GAPDH fusion protein. Mitochondrial and cytoplasmic forms are represented by red and blue, respectively

That the mitochondrial enzymes are also translated was confirmed by LC-MS/MS of extracts of *P. infestans* grown on rye media, which detected peptides corresponding to each protein. Based on quantification using the emPAI method [28], levels of cytoplasmic TPI and the mitochondrial form (from the TPI-GAPDH fusion) were similar (Fig. 5). In contrast, cytosolic GAPDH, PGK, PGM, and ENO were present at much higher levels than their mitochondrial counterparts, paralleling the results from RNA-seq. A smaller excess of cytosolic versus mitochondrial protein, 3:1, was observed for PK. However, inclusion of PPDK protein levels in the calculation increases the excess of cytoplasmic protein for interconverting phosphoenolpyruvate and pyruvate (the sum of PK and PPDK protein levels) to 10:1. Of course, metabolic activity may not parallel protein levels.

### Mitochondrial payoff-enzymes are restricted to stramenopiles

Fig. 2 summarizes the predicted subcellular location of all glycolytic enzymes in 13 eukaryotic groups, based on analyses of 72 genomes. Mitochondrial as well as cytoplasmic forms of the pay-off enzymes are present in each of three groups of oomycetes (*Phytophthora*, *Pythium*, downy mildews), each of three diatoms (*Fragilariopsis, Phaeodactylum, Thalossiosira),* and each of two brown algae *(Cladosiphon, Ectocarpus).* Many of the enzymes also have plastidic forms in brown algae, diatoms, and plants as noted previously [8, 12–14]. Brown algae and diatoms also share the TPI-GAPDH fusion protein with oomycetes, but unlike oomycetes contain a predicted mitochondrial GAPDH. While one brown alga lacks a plastid-targeted TPI, it does have a plastidic TPI-GAPDH fusion. PPDK is present only in oomycetes, diatoms, brown algae, plants, and trypanosomes. With the exception of two enzymes in the apicomplexan *Neospora caninum,* no mitochondrial glycolytic enzymes were detected in any other apicomplexan, animal, fungus, trypanosome, or slime mold.

In the three species of *Blastocystis* (*Blastocystis* subtypes 1 and 4, and *B. hominis*), both mitochondrial and cytoplasmic forms are predicted only for enolase. In contrast, the original gene models lead to TargetP and Mitofates predictions of mitochondrial PGK and cytoplasmic PGM in *B. hominis*, cytoplasmic PGK and mitochondrial PGM in *Blastocystis* subtype 4, and mitochondrial PGK and PGM in subtype 1; a predicted mitochondrial proteome of subtype 1 published while this manuscript was under review also annotated an enolase, PGK, PGM, GPDH, and TPI-GAPDH fusion as mitochondrial [29]. Interestingly, although only cytoplasmic enzymes were predicted for *B. hominis* PGM and *Blastocystis* subtype 4 PGK, short (8 and 18 amino acid) 5′ extensions of those gene models change their predicted targeting to mitochondrial. It is intriguing to consider that these genes may have alternate translation start sites, which could result in dual localization and a complete cytosolic glycolytic pathway. The importance of alternative translation start sites in eukaryotes is becoming increasingly appreciated [30].

Consistent with a prior report [8], a mitochondrial TPI-GAPDH fusion protein is predicted to be expressed by the rhizarian *Paulinella chromatophora.* We also identified a predicted mitochondrial GAPDH from that species, but neither a mitochondrial TPI-GAPDH or GAPDH was detected in the rhizarians *Bigelowiella natans, Plasmodiophora brassicae,* or *Reticulomyxa filosa.* Moreover, we found no evidence for the production of mitochondrial PGK, PGM, ENO, or PK by those species. Nevertheless, the presence of the TPI-GAPDH fusion in stramenopiles and rhizarians is intriguing in light of the proposal that they form part of the “SAR” supergroup, which unites three groups of protists [31].

It should be noted that Fig. 2 represents a consensus for each taxonomic group. Due to problematic gene models, some species first appeared to be outliers, for example when all but one species in a group had a mitochondrial form. This was usually the result of gene models that were truncated or had unsupported 5′ introns. Correcting the gene model usually restored the targeting prediction to the consensus.

### Further evidence that payoff-phase enzymes reside in mitochondria

We considered it important to confirm that the enzymes actually reside in mitochondria, due to the unusual nature of that location. Moreover, programs for predicting targeting can yield false positives and none have been tuned to stramenopiles. This was achieved by expressing PITG_00132, PITG_13749, and PITG_07405, which encode PGK, PGM, and PK, respectively, in *P. infestans* using C-terminal tdTomato or green fluorescent protein (GFP) tags. The TPI-GAPDH fusion was not tested since a prior study supported the delivery of a diatom ortholog to mitochondria [7].

**Fig. 6:**
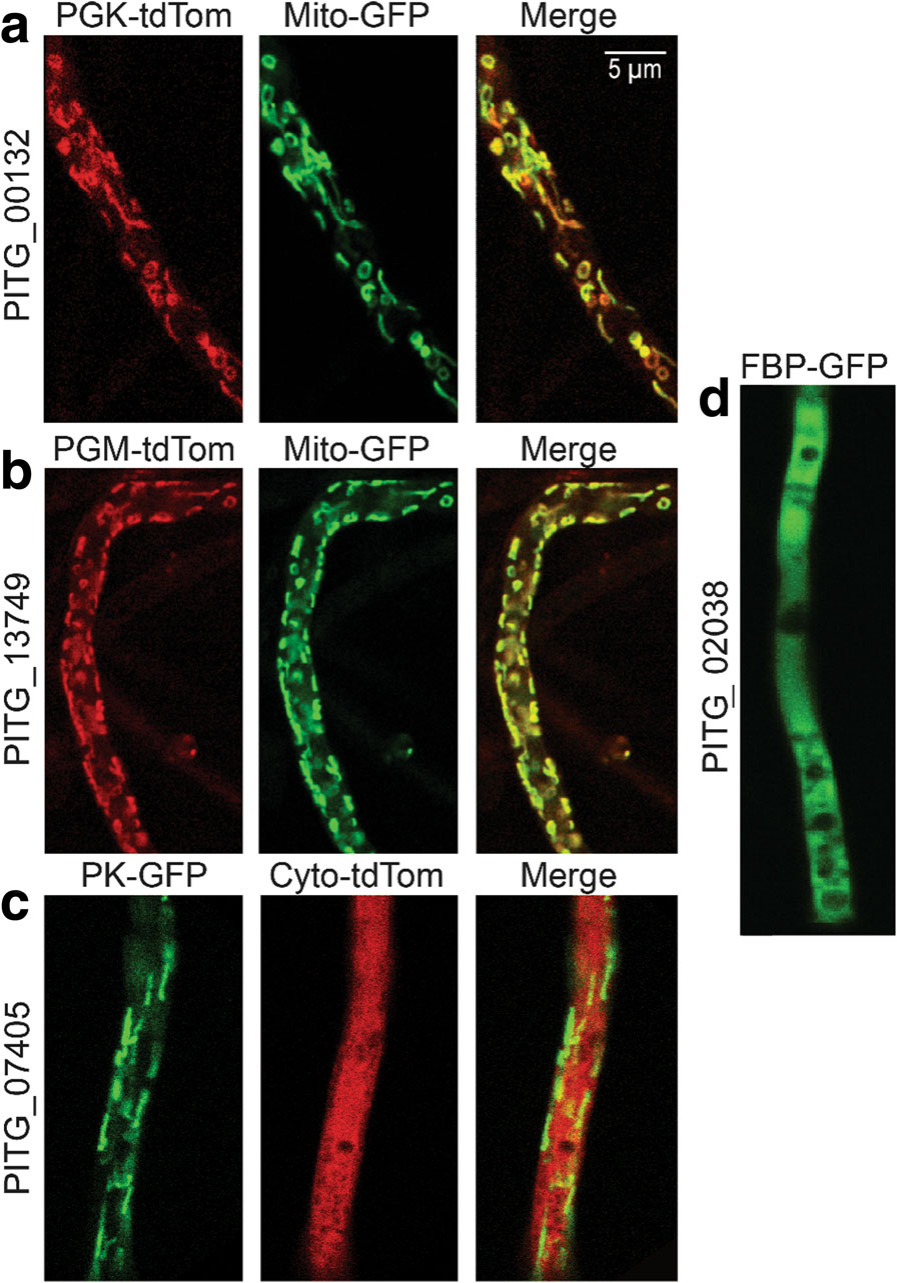
Localization of glycolytic enzymes by confocal microscopy. **a,** strain coexpressing GFP-tagged β-ATPase (Mito-GFP, green) and the PGK from gene PITG_00132 fused to tdTomato (red). **b,** coexpression of GFP-tagged β-ATPase and PGM from gene PITG_13749 fused to tdTomato. **c**, coexpression of cytoplasmic tdTomato and the PK from gene PITG_07405 fused to GFP. **d,** pattern exhibited by a canonical cytoplasmic enzyme, fructose bisphosphatase from gene PITG_02038 (FBP-GFP)

PGK, PGM, and PK were each observed to reside in mitochondria based on comparisons to a known mitochondrial protein, β-ATPase [32]. As shown in Fig. 6a and b, for example, the PGK and PGM signals were highly coincident with those of GFP- tagged β-ATPase. The images also show that mitochondria in *P. infestans* range from being round to elongated, but the enzymes did not appear to reside preferentially in organelles of any particular shape. The red/green signal ratio varied at different sites, which is consistent with observations in other taxa that mitochondria are neither biochemically or structurally uniform [33, 34]. PK also showed a pattern consistent with localization in mitochondria (Fig. 6c). As a control, we also expressed GFP fused to a cytosolic enzyme, fructose bisphosphatase (PITG_02038). This exhibited the expected cytosolic distribution, appearing throughout hyphae except for vacuolated regions which appear as dark zones (Fig. 6d).

### Oomycetes also have novel mitochondrial serine biosynthesis enzymes

**Fig. 7:**
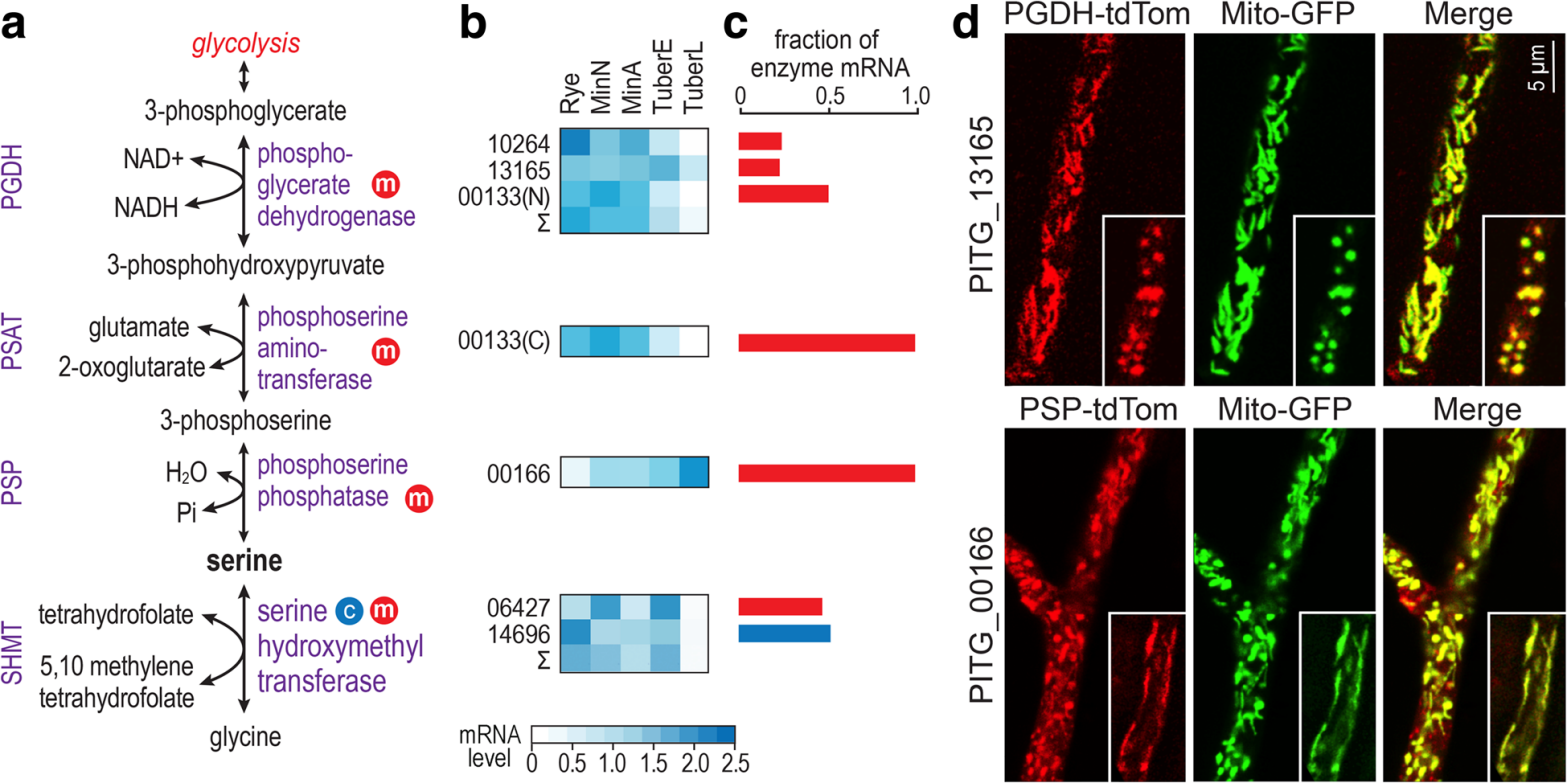
Serine biosynthesis in *P. infestans.* **a,** Enzymes for forming serine. Mitochondrial and cytosolic enzymes are marked by red and blue circlar symbols, respectively. **b,** mRNA levels in different tissues, as described in Fig. 1. For enzyme activities produced by more than one gene, the sum of CPM values in each tissue type is represented by the row labeled Σ. **c,** Contribution of each gene to the total transcript pool for each enzyme. **d,** Localization of enzymes. Top row, transformant co-expressing GFP-tagged mitochondrial marker and PGDH from PITG_00132 fused to tdTomato. Bottom, transformant co-expressing GFP-tagged mitochondrial marker and PSP from PITG_13749 fused to tdTomato. The smaller insets indicate alternative morphologies of mitochondria in *P. infestans.* The organelles are typically elongated in actively growing hyphae and rounder in dormant or slowly-growing cultures

Regardless of how oomycetes acquired their mitochondrial payoff-phase enzymes, their retention during evolution may have a physiological explanation, for example by interacting with other metabolic pathways. We therefore surveyed all 1671 *P. infestans* genes annotated as encoding metabolic enzymes for cases where the TargetP and Mitofates programs predicted mitochondrial locations, for which orthologs in most other eukaryotes were predicted or known to be cytoplasmic. This identified PGDH (EC 1.1.1.95, K00058), PSAT (EC 2.6.1.52, K00831), and PSP (EC 3.1.3.3, K01079), which comprise the so-called phosphorylated serine biosynthesis pathway (Fig. 7a). Scores in support of the mitochondrial targeting of these three enzymes from *P. infestans* and other oomycetes are recorded in Additional file 1: Table S1. This discovery is notable since the serine biosynthesis pathway is generally regarded as being cytosolic [18]. Moreover, the presence of both the serine biosynthesis and the glycolytic payoff-phase enzymes in mitochondria is significant since they are joined by a common intermediate, 3-phosphoglycerate.

PGDH in *P. infestans* is encoded by three genes, all predicted to produce mitochondrial proteins. While the PITG_10264 and PITG_13165 proteins only contain the PGDH domain, the PITG_00133 protein is a PGDH-PSAT fusion. There is no other PSAT gene in *P. infestans*. PSP is encoded by a single gene, PITG_00166.

All of the PGDH, PSAT, and PSP genes from *P. infestans* are expressed (Fig. 7b, c). Interestingly, PGDH and PSAT transcripts are higher in artificial media than on tubers, which could be a response to metabolites that are abundant *in planta.* PSP mRNA, in contrast, rises during late tuber infection, which might be a response to declining free amino acids.

The bioinformatically predicted mitochondrial targeting of the enzymes was confirmed by expressing the PITG_13165 and PITG_00166 proteins with tdTomato tags in *P. infestans* (Fig. 7d). Both colocalized with a mitochondrial marker, GFP-tagged β-ATPase. The red/green ratio varied between organelles, indicating diverse mitochondrial subpopulations as seen with the glycolytic enzymes.

As shown in Fig. 7a, *P. infestans* can also generate serine using the enzyme serine hydroxymethyltransferase, SHMT (E.C. 2.1.2.1, K00165). However, SHMT makes serine from glycine, and indirectly from other amino acids. In contrast, the PGDH-PSAT-PSP pathway makes serine de novo from the glycolytic intermediate. SHMT is predicted to exist in both mitochondrial and cytoplasmic forms in oomycetes, which are both expressed (Fig. 7b).

### A mitochondrial phosphorylated serine biosynthesis pathway is unique to stramenopiles

A summary of the predicted subcellular location of the serine enzymes in different taxa is shown in Fig. 2, beneath the corresponding data for the glycolytic enzymes. Mitochondrial PGDH, PSAT, and PSP occur exclusively in oomycetes and diatoms, which also lack the classic cytoplasmic form of the pathway. An exception is *Blastocystis*, which seems to lack PGDH, PSAT, and PSP. Oomycetes and brown algae, but not diatoms, encode PGDH and PSAT as a mitochondrial fusion protein although brown algae appear to encode only cytosolic PSP. The targeting scores for oomycetes and other stramenopiles are recorded in Additional file 1: Table S1.

In contrast, animals, fungi, slime molds, trypanosomes, apicomplexans, and rhizarians only contain the classical cytoplasmic pathway. *P. chromatophora,* the rhizarian that makes a TPI-GAPDH fusion, does not encode a PGDH-PSAT fusion. Interestingly, some apicomplexans and rhizarians entirely lack the phosphorylated pathway, and instead appear to be dependent on SHMT for generating serine. The patchy distribution of the phosphorylated pathway that we observed in apicomplexans, which represent part of the Alveolata, was also seen in other alveolate phyla. For example, while the alveolate *Symbiodinum microadriaticum* encodes predicted cytoplasmic forms of PGDH, PSAT, and PSAT, the pathway was not detected in *Paramecium tetraurelia.*

In contrast to the phosphorylated serine biosynthesis pathway, SHMT in stramenopiles has both cytoplasmic and mitochondrial forms. It is also found in the two locations in animals, fungi, and plants but is only cytoplasmic in trypanosomes, slime molds, rhizarians, and apicomplexans. Photosynthetic stramenopiles and plants can also make serine through a glyoxylate-based plastidic pathway, but not all of the corresponding enzymes can be found in oomycete genomes.

### Multiple origins of mitochondrial enzymes appear likely

**Fig. 8:**
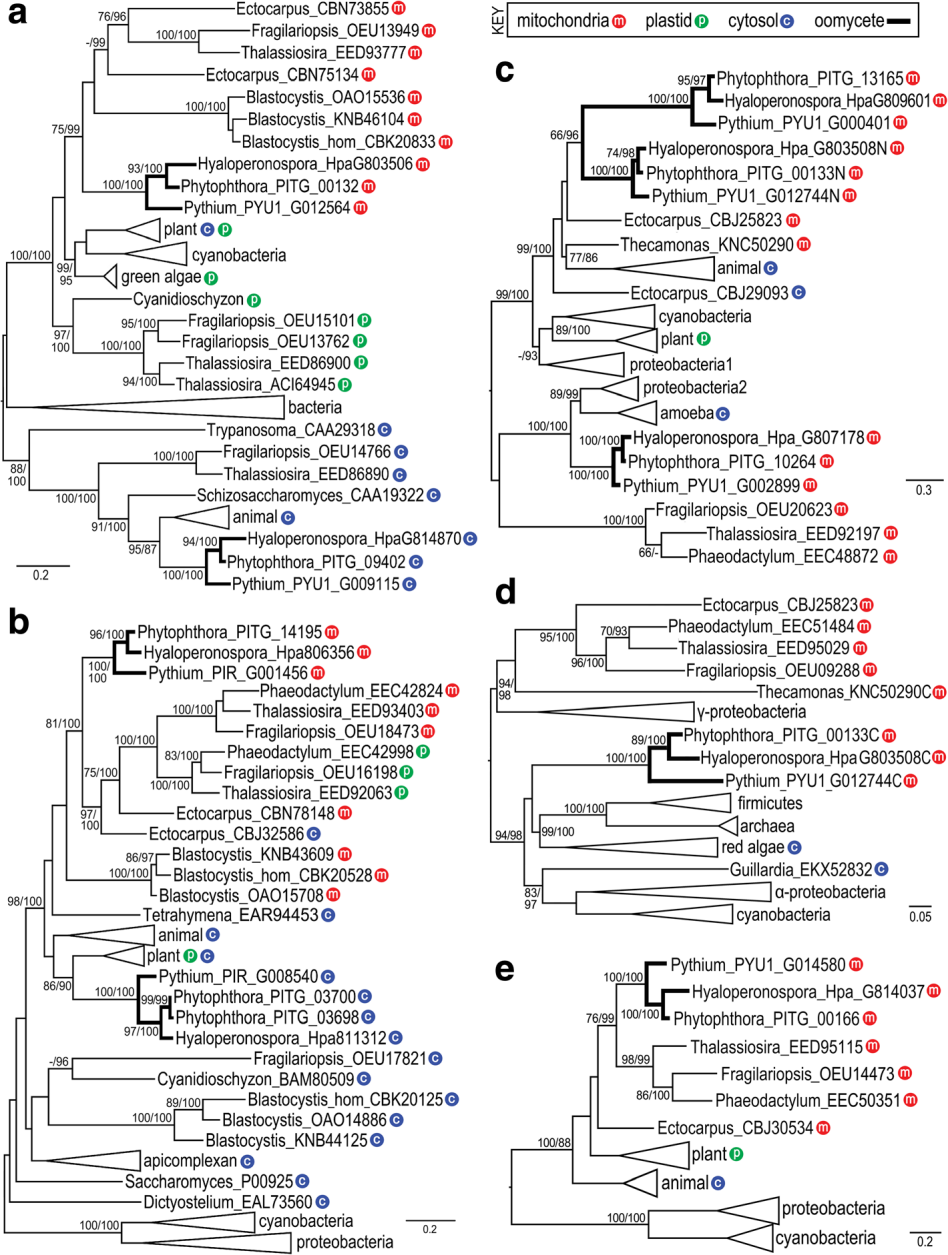
Phylogenetic analysis of selected pay-off phase glycolytic and serine biosynthesis enzymes. Shown are PhyML trees for PGK (**a**), ENO (**b**), PGDH (**c**), PSAT (**d**) and PSP (**e**). Values at nodes represent bootstrap values above 70 from PhyML, and posterior probability values above 90 from mrBayes. Oomycetes are highlighted by thick lines. Mitochondrial, cytoplasmic, and plastidic forms are represented by red, blue, and green circular symbols, respectively. PGDH-PSAT fusion proteins are represented in the PGDH tree with a N suffix (for N-terminal domain), and in the PSAT tree with a C suffix (for C-terminal domain). Not all diatoms are shown as having a cytosolic form of ENO, which is consistent with reports that some diatoms lack a complete cytosolic glycolytic pathway [13]. Species in collapsed clades and accession numbers of their proteins are in Additional file 3

Phylogenetic analyses indicated that the cytoplasmic and mitochondrial payoff-phase glycolytic enzymes of oomycetes have diverse ancestries. In the case of PGK, the mitochondrial proteins from *P. infestans* (PITG_00132 protein) and other oomycetes clustered mostly closely with the mitochondrial PGKs of diatoms and brown algae, and had additional affinity to cyanobacterial and plant enzymes (Fig. 8a). In contrast, cytosolic oomycete PGK (e.g. PITG_09402 protein) resided in a distinct clade along with cytosolic PGK from animals, fungi, and diatoms. An interesting contrast was observed between the plastidic and cytoplasmic forms of the plant and diatom enzymes. While the plant enzymes formed a single clade, the plastidic and cytoplasmic diatom enzymes formed distinct clusters associated with cyanobacteria and animals, respectively.

A somewhat distinct pattern was observed for enolase (Fig. 8b). In this case, mitochondrial oomycete ENO (e.g. PITG_14195 protein) formed a well-supported clade with diatom ENO. Interestingly, the latter included both mitochondrial and plastidic proteins, suggesting that the two forms of diatom proteins had a recent common ancestor. Occurring as a sister clade were all mitochondrial *Blastocystis* enzymes. In contrast, cytoplasmic oomycete ENO (e.g. PITG_03700 protein) formed a well-supported clade with cytoplasmic animal ENO as well as cytoplasmic and plastidic plant ENO. Cytoplasmic diatom and *Blastocystis* ENO also clustered with other cytoplasmic enzymes, although their connection to the oomycete enzymes was not as clear. One distinction between PGK and ENO was that while there was good support for the clustering of cyanobacterial PGK with the mitochondrial stramenopilian enzymes, neither form of stramenopilian ENO enzymes clustered with cyanobacterial sequences.

Complex patterns of inheritance were also suggested by phylogenetic analyses of the three serine biosynthesis enzymes. Even though all oomycete PGDHs appear to be mitochondrial, phylogenetic analyses placed the enzymes in distinct clusters (Fig. 8c). PITG_10264 protein and its oomycete orthologs clustered with amoebal and some proteobacterial proteins. In contrast, the PITG_13165 protein and the PGDH domain of brown algal and oomycete PGDH-PSAT fusion proteins (e.g. PITG_00133) clustered with animals. Also clustering with the oomycete-animal group was the PGDH domain from a PGDH-PSAT fusion from the apusozoan *Thecamonas trahens*. This was the only non-stramenopilian eukaryotic PGDH-PSAT fusion found in GenBank records.

Phylogenetic analyses of PSAT indicated patterns discrete from PGDH. All stramenopile PSAT enzymes belong to a variant form of the enzyme defined by protein domain TIGR01365 (SerC_2), which is found in a small number of distantly related species. This form lacks much affinity to the more common form of the enzyme, defined as TIGR01364 (SerC_1). In phylogenetic analyses of members of the SerC_2 family, oomycete PSAT formed a poorly-resolved cluster with orthologs from firmicutes, archaea, cyanobacteria, and α-proteobacteria (Fig. 8d). Also present in the group were red algae and the cryptophyte *Guillardia*, both of which contain plastids. In contrast, other plastid-containing stramenopiles (diatoms, brown algae) formed a separate cluster which included enzymes from *Thecamonas* and γ-proteobacteria. SerC_1 enzymes are not shown in Fig. 8d, but cluster as an outgroup to SerC_2 and include members from other bacterial groups, animals, and plants.

A different pattern was observed in analyses of the third serine biosynthesis enzyme, PSP (Fig. 8e). In this case, the oomycete PSPs formed a well-supported cluster with proteins from other stramenopiles. Some affinity was also observed between the stramenopile, plastidic plant, and animal enzymes, with bacterial enzymes appearing as an outgroup.

### Mitochondrial serine and glycolytic enzymes are encoded by neighboring genes

**Fig. 9:**
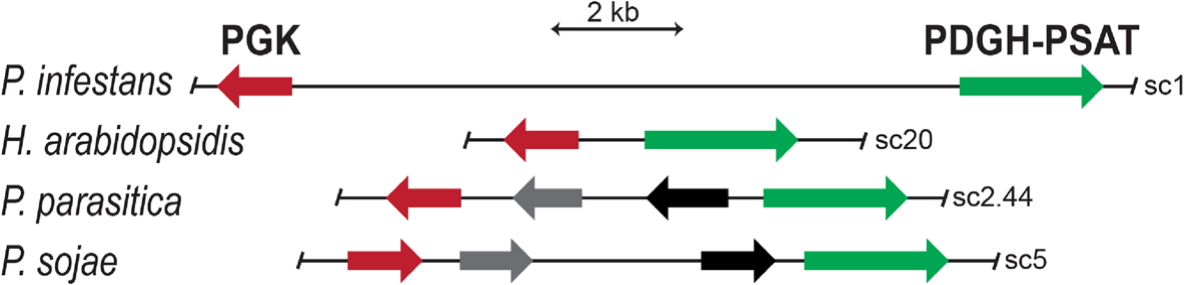
Physical linkage of genes encoding PGK and the PDGH-PSAT fusion in oomycete genomes. Black and grey arrows represent unrelated genes. Supercontig (sc) numbers are on the right

Additional insight into the origins of the unusual mitochondrial enzymes was provided by the discovery that the PGK and PDGH-PSAT genes are physically linked on oomycete chromosomes (Fig. 9). These are next to each other in *P. infestans* and *H. arabidopsidis,* although the intergenic region has expanded in *P. infestans*. In other *Phytophthora* species such as *P. parasitica* and *P. sojae* they are separated by two other genes, but are still within 6 kb of each other. The adjacency of the PGK and PDGH-PSAT genes is consistent with a shared acquisition event, although the regulatory benefits of being in a common chromatin domain may have selected for a rearrangement that joined the genes. The two genes are unlinked in other oomycetes, diatoms, and brown algae, although this is a tentative conclusion due to the fragmented nature of their genome assemblies. Interestingly, the gene encoding PSP (PITG_00166) is also physically linked to PGK and PDGH-PSAT in *P. infestans,* being 30 genes to the right of the latter (PITG_00133) on supercontig 1. This supercontig contains approximately 2.9% of the 240 Mb *P. infestans* genome [35].

## Discussion

Building on bioinformatic predictions, we used fluorescently-tagged proteins to confirm that glycolytic payoff-phase enzymes reside within *P. infestans* mitochondria. This appears needed to facilitate the transfer of 3-phosphoglycerate to the PGDH-PSAT-PSP pathway, which we demonstrate is also mitochondrial in oomycetes and not cytosolic as in most eukaryotes. Linking the two pathways is important to enable the biosynthesis of serine, and maximize the efficiency of gluconeogenesis by enabling it to use serine-derived carbon as well as pyruvate. Additional benefits of locating glycolytic enzymes in mitochondria may include raising metabolic efficiency by concentrating reactions in a smaller space [1] and eliminating a bottleneck in ATP production, which in eukaryotes with traditional metabolism is moving pyruvate from cytosol to mitochondria [36].

The same compartmentalization of glycolytic and serine metabolism enzymes that we demonstrated for *P. infestans* is also predicted for other oomycetes, diatoms, and brown algae but not *Blastocystis,* which lacks the PGDH-PSAT-PSP pathway and has only single genes encoding PGK, PGM, and ENO. Like many other animal parasites, *Blastocystis* spp. have reduced genomes in which some metabolic pathways are absent [37]. *Blastocystis* is referred to as having mitochondrion-related organelles, since only a partial Krebs cycle and oxidative phosphorylation chain are present [38]. While the main life stages of other stramenopiles contain cell walls built from gluconeogenic intermediates, only the cyst stage of *Blastocystis* contains a cell wall. This may reduce the importance of linking serine metabolism to gluconeogenesis in *Blastocystis*.

While the benefits of having glycolytic and serine pathways in the same organelle in photosynthetic stramenopiles and oomycetes may be clear, how this evolved is less apparent. One challenge in interpreting our results is that definitive information about the ancestry of stramenopile lineages are lacking [11]. It is unclear if oomycetes diverged from other stramenopiles prior to the latter’s acquisition of plastids (if oomycetes lost their plastids) or if stramenopiles experienced multiple rounds of endosymbiosis. Early studies reported finding genes of plastid ancestry in oomycetes, but the methodology of those studies have been challenged [10, 11, 39]. Nevertheless, consistent with endosymbiosis in a shared stramenopile ancestor is our observation that mitochondrial oomycete PGK and ENO cluster with their plastidic and/or mitochondrial diatom orthologs. One model is that after a shared endosymbiosis event that led to plastids, mutations converted many N-terminal plastid targeting sequences to mitochondrial import signals. The cellular machinery recognizing the two signals are distinct, but the signals themselves are similar and mutations that reduce their net charge may cause mitochondrial targeting [40, 41]. An alternative model is that plastidic and mitochondrial PGK were acquired independently, possibly by additional round(s) of endosymbiosis. The latter may have involved a red algae, in light of the close phylogenetic affinity of *Cyanidioschyzon* and plastidic stramenopile PGK.

The serine biosynthesis enzymes of stramenopiles exhibit several patterns of inheritance. Only PSP appears to have been acquired through simple descent. With PGDH, one form in oomycetes (e.g. PITG_10264) clusters with diatoms, while a distinct clade (e.g. PITG_13165) is more animal-like. In contrast, oomycete PSAT is closer to bacterial PSAT than to diatom orthologs. Horizontal transfer of PSAT to oomycetes from bacteria is possible, although not clearly demonstrated by the data. Indeed, the physical linkage of mitochondrial PGK and the PGDH-PSAT fusion, and the very existence of the PGDH-PSAT fusion, may be evidence of a more complex mode of inheritance. The physical linkage of genes acquired by lateral transfer is not uncommon in eukaryotes [42–44]. The event affecting oomycetes may have been shared with the apusozoan *Thecamonas,* which also contains a PGDH-PSAT fusion. Although most schemes suggest affinity of apusozoans to the Amoebozoa [45], it is intriguing to observe that apusozoans and stramenopiles are both biflagellates, and both encode a TPI-GAPDH fusion [8]. The PGDH-PSAT and TPI-GAPDH fusions may both benefit the cell due to increased metabolic efficiency resulting from substrate channeling [46, 47] or coregulation [48].

The replacement of the standard hexokinase of eukaryotes by a bacteria-like glucokinase in diatoms and brown algae has been described previously [13], so our discovery of the same replacement in oomycetes is not surprising. The finding is nevertheless curious since this may limit the plant sugars that can be metabolized by oomycetes, many of which are plant pathogens. Whether the glucokinases are very specific for glucose remains to be determined. This is usually the case for bacterial glucokinases, but there are exceptions [49]. In contrast, *Blastocystis* encodes only hexokinase. The order in which *Blastocystis* and oomycetes diverged from other stramenopiles is unknown, although both appear basal to diatoms and brown algae [11]. If *Blastocystis* diverged first, it is possible that a lateral transfer event occurred in the common ancestor of oomycetes and the photosynthetic stramenopiles; alternatively, a subsequent transfer event may replaced the glucokinase in *Blastocystis* during its evolution into a specialized animal parasite.

Our findings related to the glycolytic and serine metabolism enzymes raise the general question of why some pathways are cytosolic and others mitochondrial in eukaryotes. Metabolism has been shaped by both endosymbiotic and horizontal gene transfer [2, 50, 51]. Models of endosymbiosis leading to mitochondria and plastids entail engulfment of a α-proteobacterium and cyanobacterium, respectively [2, 52]. If the engulfer and engulfed were free-living, most reactions would initially be both cytosolic and organellar. However, most pathways would be retained in only one location, since gene transfer is usually a replacing event [51, 53] and there is ample evidence of the loss of much of the original α-proteobacterial and cyanobacterial components of the mitochondria and plastids during evolution [54, 55]. There is no a priori reason to assume that metabolic pathways should reside in the same location in all eukaryotes, and this premise is supported by our results.

## Conclusion

Our results shed new light on eukaryotic evolution. The analyses presented here and elsewhere [8, 56] support the evolution of stramenopilian glycolysis and serine metabolism through both additive and replacement events involving horizontal transfer, endosymbiotic transfer, and descent, resulting in a mosaic of enzymes with distinct ancestries and patterns of compartmentalization. Our results are a reminder that metabolic pathways as described in textbooks do not represent the breadth of biological diversity. We also suggest that the novel enzymes could be targets for chemicals to control *P. infestans* and relatives, which threaten global food security [57].

## Methods

### Manipulations of *P. infestans*

*P. infestans* strain 1306, isolated from a tomato field in northwest San Diego County, California USA [58] was maintained at 18 °C on rye-sucrose agar [59]. Expression studies involved centrifugation-clarified rye-sucrose broth, a defined minimal medium [60], and the latter with (NH_4_)_2_SO_4_ omitted and replaced by 1% casamino acids; cultures were inoculated with 10^4^/ml sporangia. For plant infections, tubers (cv. Russet Burbank) were washed in tap water, immersed in 10% (*v*/v) household bleach for 15 min, rinsed in water, cut into 2 mm slices, rinsed in water, and blotted dry. The slices were then placed on a metal rack 8-mm above moist towels in a box with a tight-fitting lid. For inoculating the tubers, suspensions of zoospores from 8-day cultures were adjusted to 5 ×10^5^/ml, and 0.2 ml was spread on the top of each tuber slice using a rubber policeman. Slices were kept at 18 °C in the dark and frozen in liquid nitrogen after 1.5 (early timepoint) and 4 days (late timepoint).

### Plasmid construction and transformation

Fluorescent fusion protein constructs were made using plasmids pGFPH and pTdTomatoN, which were constructed in the backbones described previously [32] except that the latter was made from pGFPN by exchanging GFP with the tdTomato gene. Target genes were amplified by polymerase chain reaction using a proofreading polymerase with primers containing the appropriate restriction sites. The fidelity of each construct was verified by DNA sequencing. The mitochondrial marker was as described [32].

Transformations of *P. infestans* were performed as described [61] using G418, hygromycin, or both as selectable markers. Transformants expressing the desired target proteins were identified by confocal microscopy (Leica TCS SP5) of hyphae from three-day old cultures. FITC and TRITC filters were used to detect GFP and tdTomato, respectively, using sequential scanning. Samples were fixed using 4% formaldehyde as described [62].

### RNA-seq analysis

Each treatment involved three biological replicates. RNA was obtained by grinding tissue to a powder under liquid nitrogen, followed by extraction using Sigma and Agilent Plant RNA kits for mycelia and tubers, respectively. Indexed libraries for sequencing were then prepared using the Illumina Truseq kit v2. Paired-end libraries were quantitated by Qubit analysis, multiplexed and sequenced on an Illumina HiSeq2500, except for the 1.5-day tuber sample which was sequenced on an Illumina NextSeq500. Data was analyzed using the systemPipeR workflow and report generation environment [63]. This included filtering and trimming reads using ShortRead, and aligning reads to the reference genome [35] using Bowtie 2.2.5 and Tophat 2.0.14, allowing for one mismatch. Expression calls were made with edgeR [64] using TMM normalization, a generalized linear model, and FDR calculations based on the Benjamini-Hochberg method. Differential expression calls were made based on a FDR cut-off of 0.05. Heatmaps were generated using the TMM-normalized CPM values using Partek Genomics Suite.

### Sequence retrieval and predictions of targeting

Protein sequences of the enzymes were obtained through a combination of keyword searches and BlastP analyses starting from the *P. infestans* sequences. In general, sequences were obtained from Ensemble Protists (protists.ensembl.org, release 35), Uniprot (uniprot.org; release 2017_04), PlasmoDB (plasmodb.org, release 32), TriTrypDB (tritrypdb.org, release 32), FungiDB (fungidb.org, release 32), the Joint Genome Institute (phytozome.jgi.doe.gov, release 12, or genome.jgi.doe.gov for *T. pseudonana* v. 3, *F. cylindricus* v. 1, and *P. tricornatum* v. 2), or in the case of *Cladosiphon okamuranus from* marinegenomics.oist.jp [65]. Additional sequences, particularly from bacteria, were obtained through BlastP searches of Genbank (releases 219 and 220). For stramenopiles, TBlastN was used to search for unannotated genes in each genome. The function of each sequence were confirmed by checking for the appropriate domain using the Conserved Domain Database search engine [66]. Sequences from brown algae and diatom genomes, which may not have been pure cultures, were screened for contaminating sequences; several genes showing >99% identity to marine bacteria were discarded.

Targeting predictions were made using TargetP and Mitofates [26, 27] for species lacking plastids, using cutoff and relative confidence scores of 0.85 and 2, respectively. Plant sequences were analyzed using TargetP and ChlorP [26]. Sequences from diatoms and brown algae were evaluated using Hectar [67] and ASAFind [68]. In some cases, the results were compared to those of Wolf PSORT, iPSORT, and PredAlgo in an attempt to reach consensus [69–71]. When sequences from a species appeared to be outliers, or the proteins did not start with methionine, the 5′ ends of each gene model were evaluated and corrected. In most cases, this involved a combination of comparisons to orthologs and predicting genes using GENSCAN [72]. In selected cases, RNA-seq data from GenBank’s Short Read Archive were used to help identify the 5′ end of the transcript.

Predictions of targeting across eukaryotic groups (Fig. 2) involved four *Phytophthora*, five *Pythium*, two downy mildew, three diatom, two brown algae, ten apicomplexans, three fungi, one slime mold, six trypanosomes, 35 plants, and four rhizarians. The species are listed in Additional file 2.

### Phylogenetic analysis

Sequences were obtained as described in the prior section, and checked for the presence of the appropriate protein domain using the Conserved Domain Database [65]. Protein alignments were made using MUSCLE [73] and refined using TCS [74]. The latter removed gaps and uninformative or unstable columns from the alignment, using minimal and maximum filtering options of 4 and 9, respectively. This resulted in alignments of 248, 343, 394, 429, 446, 363, and 213 amino acids for GK, PFK, PGK, ENO, PGDH, PSAT, and PSP, respectively. Prior to tree-building, substitution models were compared using ProtTest [75]. Trees were then generated using PhyML using the LG substitution model using 500 nonparametric bootstrap replicates, four rate categories, the estimated gamma distribution parameter, and the optimized starting BIONJ tree. Similar relationships between oomycete and non-oomycete proteins were drawn when considering Shimodaira-Hasegawa-like aLRT values as a measure of branch support. Trees shown in the figures were developed using PhyML with midpoint rooting. Trees were also generated using MrBayes 3.6 [76], using 500,000 generations, sampling every 200 cycles, 125,000 burn-in cycles, gamma distributed variation, and four heated chains. Accession numbers of genes used for generating the trees are shown in the figures or in Additional file 2.

### Proteomics

Proteins were extracted from hyphae grown in rye broth by bead-beading in 20 mM Tris-HCl pH 8.0, 150 mM NaCl, 10 mM EDTA, pH 8.0, 0.2% NP-40, 0.02 mg/ml heparin, 1.5 mM DTT, 1 mM PMSF, 20 units/ml DNase I), and clarified by centrifugation at 20,000 x *g* for 10 min. Protein (100 mg) was separated by 10% acrylamide SDS-polyacrylamide gel electrophoresis, gel slices (1 mm-squares) were treated for 1 h at 60 °C with tris-(2-carboxyethyl)-phosphine, and then incubated with trypsin at at 37 °C overnight. The slices were then equilibrated in 5% acetonitrile and 0.1% trifluoroacetic acid, vortexed for 15 min, mixed with the solution from the trypsin digest, and the liquified material was reduced to 10 μl under vacuum. Separations were then made using the multidimensional protein identification (MudPIT) approach as described [77]. MS/MS spectra were evaluated with MASCOT 2.1 [78] and searched against sequences in the *P. infestans* protein database. The search was configured to assume a tryptic digest, one peptide with 95% confidence, and up to one missed cleavage per peptide. Monoisotopic mass values were used, with peptide mass tolerance and fragment mass tolerance set at 60 ppm and 0.2 Da, respectively, and a cut-off value MASCOT score of 50. Quantification was performed using the emPAI approach [28].

## Additional files

Additional file 1: Table S1. Mitochondrial targeting scores of enzymes. (XLS 66 kb)

Additional file 2: Species represented in Fig. 2 and accession numbers of the analyzed protein sequences. (XLSX 43 kb)

Additional file 3: Accession numbers of protein sequences used in phylogenetic analyses of payoff phase glycolytic enzymes and serine biosynthesis enzymes. (DOCX 177 kb)

## Abbreviations

CPM: Counts per million mapped reads
EC: Enzyme Commission number
ENO: Phosphopyruvate hydratase (enolase)
GAPDH: Glyceraldehyde phosphate dehydrogenase
GFP: Green fluorescent protein
PFK: Phosphofructokinase
PGDH: Phosphoglycerate dehydrogenase
PGK: Phosphoglycerate kinase
PGM: Phosphoglycerate mutase
PK: Pyruvate kinase
PPDK: Pyruvate phosphate dikinase
PPi: Pyrophosphate
PSAT: Phosphoserine aminotransferase
PSP: Phosphoserine phosphatase
SHMT: Serine hydroxymethyltransferase
TPI: Triosephosphate isomerase
