# Supplementary material for: Rethinking the evolution of eukaryotic metabolism: novel cellular partitioning of enzymes in stramenopiles links serine biosynthesis to glycolysis in mitochondria
Source: BMC Evol Biol. 2017 Dec 4;17:241. doi: 10.1186/s12862-017-1087-8 (PMC5715807; doi:10.1186/s12862-017-1087-8)
Supplement: Supplementary file 3 — Accession numbers of protein sequences used in phylogenetic analyses of payoff phase glycolytic enzymes and serine biosynthesis enzymes. (DOCX 177 kb) [file 12862_2017_1087_MOESM3_ESM.docx]

**ADDITIONAL DATA FILE 3**

**Sequences used for PGK tree.** Accession numbers are shown in Fig. 8, except for collapsed clades. Sequences in collapsed clades are as follows: The collapsed bacterial clade includes *Acidothermus cellulolyticus* ABK52887.1; *Clostridium papyrosolvens* EGD49746.1; *Escherichia coli* CAA32604.1; *Geobacillus stearothermophilus* P18912; *Mycobacterium smegmati* ABK71882.1; *Acetivibrio cellulolyticus* WP_010251554.1; *Pseudobacteroides cellulosolvens* KNY28480; *Campylobacter concisus* OEY23938.1; *Desulfuromonas* sp. TF WP_027714084.1; *Mesorhizobium* sp. F7 WP_047564702.1; and *Pelobacter* sp. SFB93 APG26829. The cyanobacteria clade includes *Cyanobacterium aponinum*, AFZ53156.1; *Moorea producens,* AOY83283.1; *Prochlorococcus marinus*, WP_075537253.1; *Rivularia* sp. WP_015119342.1; and *Synechococcus* sp. WP_026101118.1. The animal clade includes *Castor canadensis,* JAV43119.1; *Bos taurus,* XP_005888733.1; and *Homo sapiens,* P00558, and *Brugia malayi,* CRZ23090. The plant clade includes *Arabidopsis thaliana* NP_176015.1, NP_187884.1, and NP_178073.1; *Solanum lycopersicum*, XM_004243920.2*,* XM_004246588.3, and NM_001329591.1; *Nicotiana sylvestris* XP_009763674.1; *Solanum lycopersicum*, XM_004243920.2*,* XM_004246588.3, NM_001329591.1; and *Zea mays* NP_001142404.1. The green algae include *Chlamydomonas reinhardtii,* EDO98586; and *Volvox carteri,* XP_002951648.

**Sequences used for ENO tree.** Accession numbers are shown in Fig. 8, except for collapsed clades. Sequences in collapsed clades are as follows: Animal sequences are *Gallus gallus* NP_990450.1; *Drosophila melanogaster* CAA34895.1; *Caenorhabditis elegans* NP_001022349.1; and *Homo sapiens* NP_001419.1. Plant sequences are *Glycine max* XP 003521438, *Nicotiana attenuat*a XP 019229421; *Oryza sativa* XP_015614256, XP_015632611, XP 015643741; *Solanum lycopersicum* NP_001234080, *Solanum pennellii* NP 001332774, *Arabidopsis thaliana* NP_181192, and *Chlamydomonas reinhardtii* EDO96709.1; Apicomplexan sequences are *Neospora caninum* XP_003884000.1, XP_003883999.1; and *Plasmodium knowlesi* XP_002258802.1. Cyanobacterial sequences are *Synechococcus* sp. WP_011934266.1; *Prochlorococcus marinu*s WP_012007014.1; and *Gloeobacter violaceus* WP_011142119.1. Proteobacterial sequences are *Xanthomonas oryzae* AAW76217.1; *Yersinia pestis* WP_016678559.1; *Pseudomonas fluorescens* WP_011332697.1; *Pectobacterium carotovorum* C6DDJ5; and Nostoc sp. PCC 7120] WP_010997688.1

**Sequences used for PGDH tree.** Accession numbers are shown in Fig. 8, except for collapsed clades. Sequences in collapsed clades are as follows: Proteobacteria includes *Blastomonas* sp. CCH1-A6, WP_066283978.1; Deltaproteobacteria bacterium, OGQ83248.1; Proteobacteria bacterium SG_bin5, OQW38611.1; *Rhizobium* sp. OK665, WP_037099842.1; *Sphingomonas* sp. NFR15, SDA35253.1. *Methanocaldococcus* sp. FS406, ADC70302; *Thermodesulfovibrio* sp. N1, ODA43708; *Aquaspirillum serpens,* WP_022653946.1; *Pseudomonas aeruginosa,* WP_033833465.1; *Shewanella violacea,* BAJ03636.1; and *Xanthomonas arboricola,* WP_047125460.1. Cyanobacterial PGDHs are from *Fischerella* sp. PCC 9605, WP_026731560.1; *Microcystis aeruginosa,* WP_002784892.1; *Nostoc piscinale,* WP_062294450.1; and *Oscillatoriales cyanobacterium,* OCQ92606.1. Amoebae include *Acanthamoeba castellanii,* AGX13814.1; and *Dictyostelium discoideum,* EAL66832.1. Animals include *Gallus gallus,* XP_422226.3; *Homo sapiens,* AF171235; *Mus* *musculus,* BAC36494.1 ; *Octopus bimaculoides,* XP_014790629; *Rattus norvegicus,* AAH86327. Plants include *Arabidopsis thaliana* NP_001031061.2, NP_195146.1; NP_566637.2; *Oryzae sativa* BAF16008.1, BAD09434.1.; *Physcomitrella patens* EDQ68443.1; and *Volvox carteri* XP_002950373.1. Also included is the N-terminal PGDH domain from the apusozoan *Thecamonas trahens*, XP_013756837.1.

**Sequences used for PSAT tree.** Accession numbers are shown in Fig. 8, except for collapsed clades. Sequences in collapsed clades are as follows: Cyanobacteria include *Acaryochloris marina,* ABW27168.1; *Stanieria cyanosphaera,* AFZ34742.1; *Nostoc* sp., BAT54662.1; *Calothrix parasitica,* BAY83308.1; *Rivularia* sp. PCC 7116, WP_015119757.1; and *Alkalinema* sp. CACIAM; OUC14157.1.

Gamma-proteobacteria include *Simiduia agarivorans,* WP_015046089.1; *Paraglaciecola arctica,* WP_007624213.1; *Catenovulum agarivorans,* WP_035015076.1; *Vibrio shilonii,* WP_088877193.1; *Psychromonas hadalis,* WP_022941820.1; *Gilvimarinus agarilyticus,* WP_041523585.1; *Oleiphilus messinensis,* WP_087459730.1; *Hahella* sp. CCB-MM4, WP_094705555.1; *Moritella viscosa,* CED62205.1; and *Phyllobacterium* sp. CL33Tsu, SFI81455.1.

Alpha-proteobacteria include *Mesorhizobium* sp. AA23, WP_067319442.1; *Ochrobactrum* sp. A44, ASV86305.1; *Paramesorhizobium deserti,* WP_068883045.1; *Brucella melitensis,* ARY69462.1; and *Rhizobiales* bacterium 63-22, OJY03720.1. Firmicutes include *Clostridium clariflavum,* WP_014256274.1; *Ruminiclostridium thermocellum,* ABN51533.1; *Acetivibrio cellulolyticus,* WP_010248814.1; and *Pseudobacteroides cellulosolvens* WP_036935748.1. Also shown are the red alga *Cyanidioschyzon merolae,* BAM83229.1; *Galdieria sulphuraria*, XP_005705129.1; and *Chondrus crispus,* XP_005713038.1. Note that the gene model in Genbank for PITG_00133 has a false C-terminal extension, which was corrected for this analysis as shown in Table S1.

**Sequences used for PSP tree.** Accession numbers are shown in Fig. 8, except for collapsed clades. Sequences in collapsed clades are as follows: The plant clade contains *Arabidopsis thaliana,* NP_973858.1; *Brachypodium stacei,* XP_003576098.1 and XP_003577451.1; *Brassica oleracea,* XP_013604133.1, XP_013683033.1, and XP_013638420.1; *Oryzae sativa* XP_015615356.1 and XP_015619367.1; Selaginella moellendorffii, XP_002972198.1; and *Physcomitrella patens*, EFJ27115.1. Proteobacteria include Proteobacteria bacterium ST_bin1, OQW73800.1; *Methylomicrobium alcaliphilum,* WP_014147525.1; *Methylosarcina fibrata,* WP_020565838.1; *Methylomonas methanica*, WP_064010592.1, and Methylococcaceae bacterium NSP11, OYV16031.1. The cyanobacterial clade contains *Calothrix parasitica,* BAY81408.1; *Mastigocoleus testarum,* KST63157.1; *Richelia intracellularis,* CDN15017.1; and *Rivularia* sp., AFY56867.1. Animals are *Homo sapiens,* NP_004568.2; *Python bivittatus* XP_007429749.1; *Rattus norvegicus,* AAH88310.1 and *Xenopus laevis,* XP_018100592.1.
